# Licensing of Primordial Germ Cells for Gametogenesis Depends on Genital Ridge Signaling

**DOI:** 10.1371/journal.pgen.1005019

**Published:** 2015-03-04

**Authors:** Yueh-Chiang Hu, Peter K. Nicholls, Y. Q. Shirleen Soh, Joseph R. Daniele, Jan Philipp Junker, Alexander van Oudenaarden, David C. Page

**Affiliations:** 1 Whitehead Institute, Cambridge, Massachusetts, United States of America; 2 Department of Biology, Massachusetts Institute of Technology, Cambridge, Massachusetts, United States of America; 3 Department of Physics, Massachusetts Institute of Technology, Cambridge, Massachusetts, United States of America; 4 Hubrecht Institute—KNAW (Royal Netherlands Academy of Arts and Sciences) and University Medical Center Utrecht, Utrecht, Netherlands; 5 Howard Hughes Medical Institute, Whitehead Institute, Cambridge, Massachusetts, United States of America; Stowers Institute for Medical Research, UNITED STATES

## Abstract

In mouse embryos at mid-gestation, primordial germ cells (PGCs) undergo licensing to become gametogenesis-competent cells (GCCs), gaining the capacity for meiotic initiation and sexual differentiation. GCCs then initiate either oogenesis or spermatogenesis in response to gonadal cues. Germ cell licensing has been considered to be a cell-autonomous and gonad-independent event, based on observations that some PGCs, having migrated not to the gonad but to the adrenal gland, nonetheless enter meiosis in a time frame parallel to ovarian germ cells -- and do so regardless of the sex of the embryo. Here we test the hypothesis that germ cell licensing is cell-autonomous by examining the fate of PGCs in *Gata4* conditional mutant (*Gata4* cKO) mouse embryos. *Gata4*, which is expressed only in somatic cells, is known to be required for genital ridge initiation. PGCs in *Gata4* cKO mutants migrated to the area where the genital ridge, the precursor of the gonad, would ordinarily be formed. However, these germ cells did not undergo licensing and instead retained characteristics of PGCs. Our results indicate that licensing is not purely cell-autonomous but is induced by the somatic genital ridge.

## Author Summary

During embryonic development, stem cell-like primordial germ cells travel across the developing embryo to the genital ridge, which gives rise to the gonad. Around the time of their arrival, the primordial germ cells gain the capacity to undertake sexual specialization and meiosis—a process called germ cell licensing. Based on the observation that meiosis and sexual differentiation can occur when primordial germ cells stray into the area of the adrenal gland, the primordial germ cell has been thought to be responsible for its own licensing. We tested this notion by examining the licensing process in mutant mouse embryos that did not form a genital ridge. We discovered that in the absence of the genital ridge, primordial germ cells migrate across the developing embryo properly, but instead of undergoing licensing, these cells retain their primordial germ cell characteristics. We conclude that licensing of embryonic primordial germ cells for gametogenesis is dependent on signaling from the genital ridge.

## Introduction

In mammals, both the testis and ovary derive from a common precursor structure, the bipotential gonad [1]. The development of the bipotential gonad involves two simultaneously occurring processes. The coelomic epithelium on the ventromedial surface of the mesonephros transforms from a monolayer into a thickened, multilayer epithelial structure, the genital ridge. Meanwhile, primordial germ cells (PGCs) that have migrated from the base of the allantois start arriving at the genital ridge, as early as the monolayer stage, and multiply as the genital ridge thickens. The formation of the bipotential gonad in mouse embryos begins at embryonic (E) day 10.0 and continues until E11.5-E12.0, when sexual differentiation takes place [2–4].

Migratory PGCs maintain a genomic program associated with pluripotency [5,6]. They express core pluripotency genes (*Oct4*, *Nanog*, and *Sox2*) and are able to form teratomas following their injection into postnatal mouse testes [7]. Around the time of their arrival at the genital ridge, PGCs undergo a global change in gene expression [8–10]. Specifically, the PGCs turn on a set of genes that enable them to undergo sexual differentiation and gametogenesis, and to switch off their pluripotency program. Following this transition, germ cells are referred to as gametogenesis-competent cells (GCCs), and are poised to initiate meiosis as well as male or female differentiation [11–13]. Upon the development of the genital ridge into either a testis or an ovary (at ~E12.5 in mouse embryos), GCCs respond to cues from the somatic environment and enter either the spermatogenic or oogenic pathway accordingly. The transition from PGC to GCC is referred to as germ cell licensing [11], and it represents a critical transformation of germ cells to a sexually competent state.

One of the genes upregulated in germ cells at the time of licensing is *Dazl*, which encodes an evolutionarily conserved and germ-cell-specific RNA-binding protein [14]. In mouse embryos of C57BL/6 genetic background, germ cell licensing is dependent on *Dazl* [11,15]. In *Dazl-*null embryos, germ cells retain characteristics of PGCs and fail to embark upon the pathways to oogenesis or spermatogenesis in the fetal ovary or testis, respectively. However, what triggers *Dazl* expression and germ cell licensing remains unknown.

One hypothesis, based on observational studies, states that licensing is triggered in a cell-autonomous and gonad-independent manner. As PGCs migrate to the genital ridge, a fraction of them are left in places along the migratory path, such as in the allantois, tail, midline, spinal cord, and adrenal gland [16,17]. While most of these ectopic PGCs die, those migrating to the adrenal gland survive until ~3 weeks after birth [16,18–20]. Upadhyay and Zamboni [19] observed that these adrenal germ cells, regardless of the sex of the fetus, enter meiosis according to the schedule of normal ovarian germ cell development. Based on these findings, the authors hypothesized that PGCs transition into meiotic germ cells (oocytes) in a gonad-independent, and therefore cell-autonomous, manner. This hypothesis was further supported by several *in vitro* studies [13,21–23], showing, for instance, that PGCs isolated from E10.5 mouse embryos of both sexes continue to develop *in vitro* and initiate meiosis at approximately the same time as meiotic entry occurs *in vivo* [13,22,23].

Previous studies from our lab and others led us to question this hypothesis and suggest an alternative: PGCs undergo germ cell licensing in response to external signals, upon migration to the genital ridge. The authors who proposed the cell-autonomous hypothesis considered E10.5 PGCs to be pre-gonadal germ cells [22,23]. However, we recently showed that the marker of genital ridge formation, GATA4, is expressed as early as E10.0 [2]. It is plausible that the E10.5 PGCs used in the *in vitro* studies had already been exposed to gonadal factors. In addition, the claim that the PGCs in the adrenal gland transition to meiotic germ cells without exposure to the genital ridge belies the fact that the adrenal anlagen and genital ridge derive from a common precursor, called the adrenogonadal primordium. These two organs are not segregated completely until ~E11.5 [24,25]. Adrenal PGCs would therefore be exposed to the genital ridge, or its equivalent, during a short interval in their development. These findings raise doubts about whether the transition of PGCs to meiosis-competent cells is gonad-independent, or induced by factors shared by the developing gonad and adrenal gland.

Germ cell licensing precedes meiotic entry [11,15]. Since the occurrence of licensing coincides with the arrival of PGCs at the genital ridge, we suspected that the genital ridge provides extrinsic signals required for inducing germ cell licensing. Initiation of genital ridge formation depends on the transcription factor GATA4, which is expressed in the somatic compartment, but not in germ cells [2]. We therefore utilized *Gata4* conditional knockout (cKO) embryos, which lack the genital ridge, to test the hypothesis of genital ridge-dependent licensing. If true, we would expect that in the absence of the genital ridge, PGCs would fail to undergo licensing and subsequent meiotic entry. The result of this study would provide fundamental insight into how germ cells switch off their pluripotency program and acquire competence for meiosis and sexual differentiation.

## Results

### Anterior-to-posterior expression of the germ cell licensing marker *Dazl*

**Fig 1:**
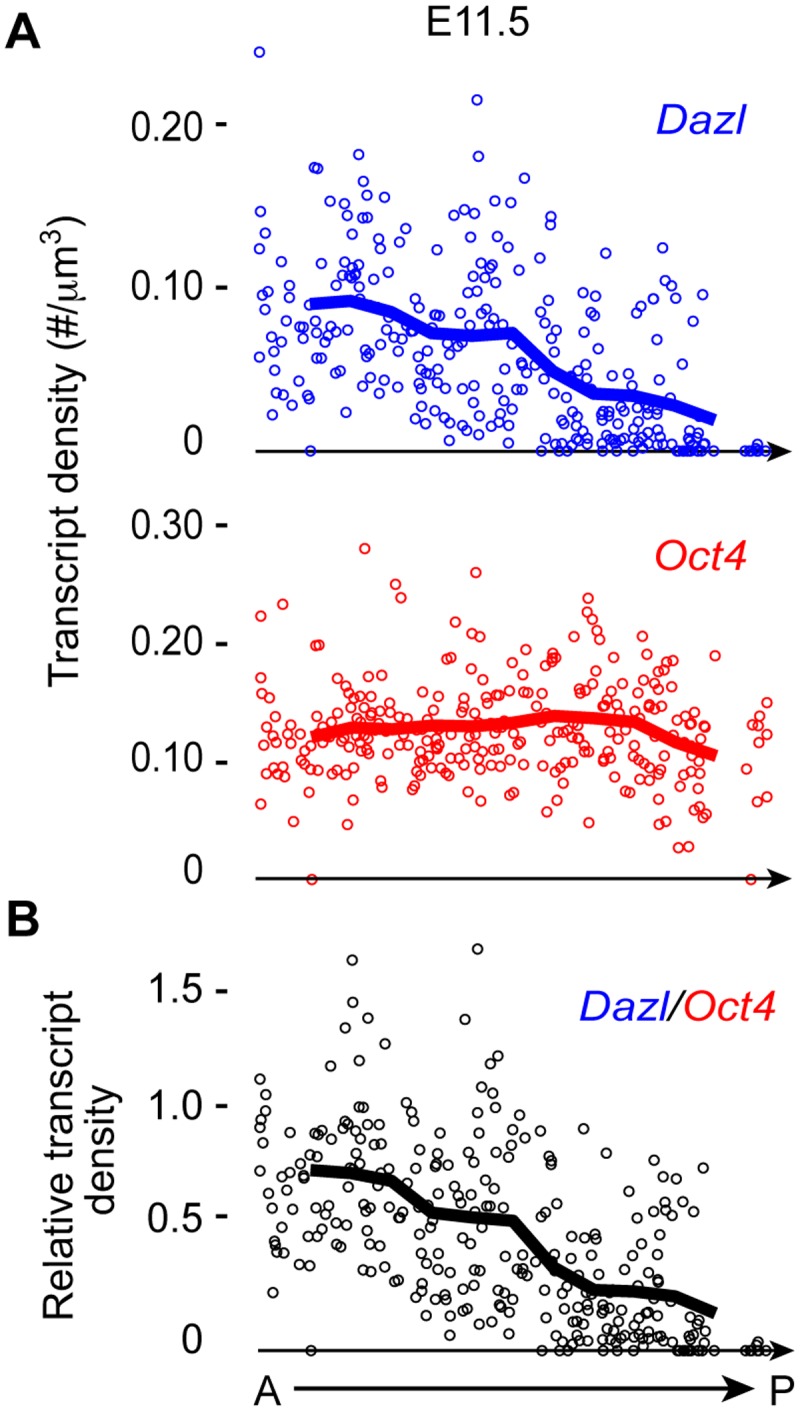
*Dazl* expression in germ cells displays an A-P gradient along the genital ridge. (A) Scatterplots represent transcript densities of *Dazl* and *Oct4* in individual germ cells along A-P axis of genital ridge of E11.5 embryos (n = 3), as measured by smFISH. (B) *Dazl* transcript density was normalized against *Oct4* transcript density for each individual cell to obtain relative *Dazl*:*Oct4* transcript density per cell. Lines in plots represent average transcript density of cells (A) or ratio of densities (B) at a particular A-P position.

The genital ridge develops in an anterior-to-posterior (A-P) direction starting at E10.0 [1,2], as PGCs are entering the region. *Dazl* is expressed in germ cells during licensing for gametogenesis [8,9,11,14,15]. If the genital ridge regulates germ cell licensing, we would expect to find a similar A-P induction of licensing, along with *Dazl* expression. To test this prediction, we quantified *Dazl* transcript levels in individual germ cells along the A-P axis of the genital ridge using single-molecule fluorescence *in situ* hybridization (smFISH) [26]. We first confirmed that *Dazl* expression was below the detectable level in migratory PGCs at E9.5, as expected (S1A Fig). When examining post-migratory germ cells at E11.5, we detected a gradient of *Dazl* transcript levels along the genital ridge; expression is highest in the anterior portion and decreases in an A-P direction (Figs. 1 and S1B). As a comparison, we also quantified the transcript level of *Oct4* (*Pou5f1*), a pluripotency gene that is expressed in both migratory PGCs and post-migratory germ cells. We did not observe an A-P gradient of *Oct4* transcript levels in germ cells. Instead, germ cell *Oct4* transcript levels were relatively consistent along the length of the genital ridge. Therefore, the A-P expression pattern of *Dazl* supports the possibility that the genital ridge induces germ cell licensing.

### Genital ridge formation is required for germ cell licensing

**Fig 2:**
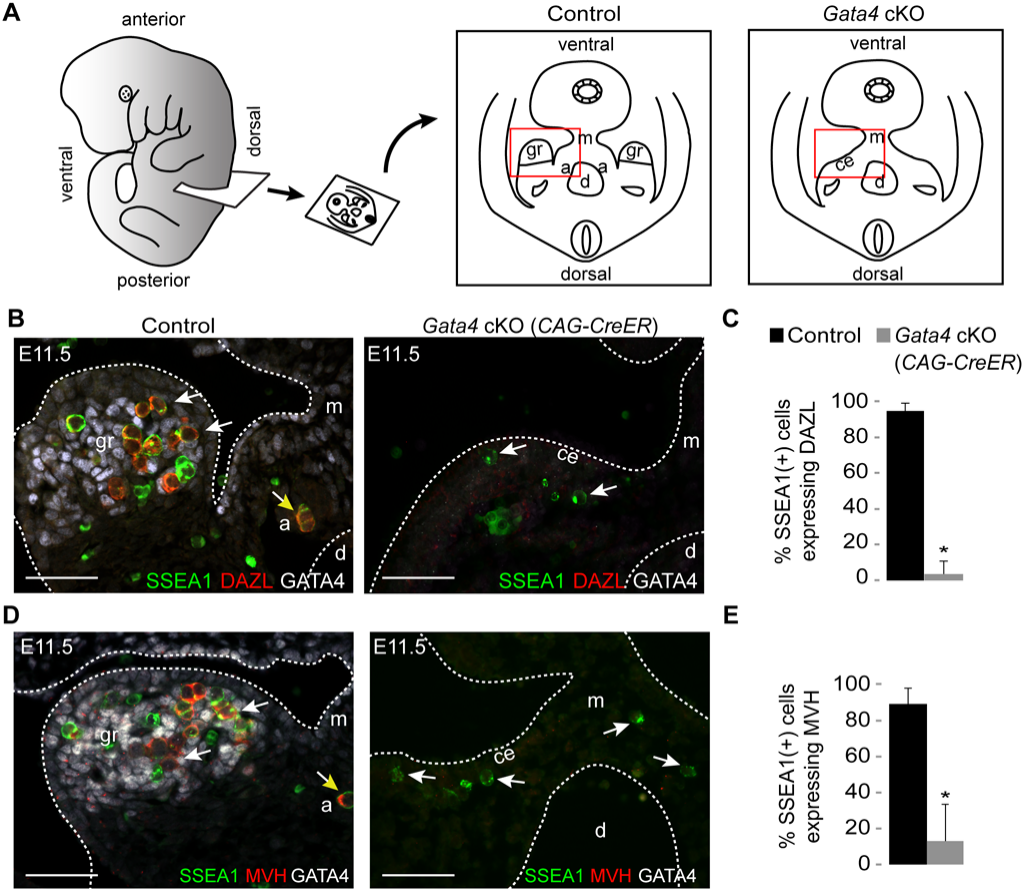
Germ cells in *Gata4* cKO embryos do not express DAZL or MVH. (A) Schematic illustration of transverse section through embryo trunk that contains urogenital ridge. Red boxes indicate areas imaged, shown in B and D. a, adrenal gland; ce, coelomic epithelium; d, dorsal aorta; gr, genital ridge; m, mesentery. (B and D) Immunofluorescent staining for SSEA1, DAZL, MVH, and GATA4 protein in transverse sections of control (*Gata4* ^*+/flox*^ *)* and *Gata4* ubiquitous cKO (*CAG-CreER*) embryos on a mixed genetic background. Representative germ cells are indicated by white arrows. Germ cells mis-migrating to the adrenal gland (yellow arrows) also express DAZL and MVH. Scale bars: 50 μm. (C and E) Percentage of germ cells that are positive for DAZL or MVH expression in control and *Gata4* cKO (*CAG-CreER*) embryos. SSEA1 marks all germ cells at this age. Plotted here are means ± standard deviation from biological replicates (n ≥ 3 for each genotype). *, *P* < 0.05 (two-tailed Student’s *t*-test).

We next asked whether germ cell licensing requires the presence of the genital ridge. We examined DAZL expression in transverse sections of E11.5 embryos in which we had prevented genital ridge formation by ubiquitous deletion of *Gata4* (*Gata4* cKO) through tamoxifen injection at E8.75 (Fig. 2A, *Gata4* ^*flox/∆*^ *; CAG-CreER* [2]). Sections were immunostained for SSEA1, DAZL, and GATA4 expression. SSEA1 was used to identify all germ cells at this time point, as it identifies both migratory PGCs and post-migratory germ cells. In sections from littermate controls (*Gata4* ^*+/flox*^ *)*, we found that the majority of germ cells that had colonized the genital ridge expressed DAZL, consistent with our smFISH data (Fig. 1). In contrast, germ cells in *Gata4* cKO embryos migrated to the ventromedial side of the mesonephros (the location of the genital ridge in wild type animals) but failed to initiate DAZL expression (95% vs. 4%, respectively; Fig. 2B and C). These results indicate that genital ridge formation is essential for DAZL expression in PGCs, but not for their migration.

Like *Dazl*, *Mouse vasa homolog* (*Mvh*, also known as *Ddx4*) is also expressed in germ cells around the time of their arrival at the genital ridge [27]. In *Dazl*-null embryos of the C57BL/6 genetic background, germ cells do not undergo licensing, but MVH is still expressed in these cells, suggesting that MVH expression is independent of *Dazl* [11]. We then examined MVH expression in the germ cells of *Gata4* cKO embryos. In transverse sections from littermate controls, the majority of germ cells that had colonized the genital ridge expressed MVH, whereas germ cells in *Gata4* cKO embryos failed to express MVH (89% vs. 13%; Fig. 2D and E). These results indicate that, like DAZL, MVH expression is also dependent upon genital ridge formation.

To establish that the failure of germ cell licensing was due to the loss of *Gata4* in somatic tissues, we used an additional Cre line, *Osr1-CreER*, that is expressed in genital ridge (somatic) precursor cells but not in the germ line. As with the ubiquitous *Gata4* cKO described above, *Osr1*-driven loss of *Gata4* in the soma resulted in PGCs migrating to the ventromedial side of the mesonephros, without initiating either DAZL or MVH expression (S2 Fig). These results confirm that licensing of germ cells depends upon *Gata4* function in somatic cells.

Consistent with previous observations that mis-migrated adrenal germ cells can initiate meiosis, we noticed that germ cells that had migrated to the adrenal gland expressed DAZL and MVH at E11.5 (Fig. 2B and D). These findings suggest that germ cells in the adrenal gland undergo licensing as they do in the genital ridge, which makes germ cells there capable of entering meiosis [19,20]. Licensing of the adrenal germ cells is likely to be dependent on factors shared between the genital ridge and the adrenal, as both organs derive from the same primordium.

To confirm that genital ridge formation is sufficient to induce the germ cell licensing factor DAZL, we examined embryos lacking either *Wt1* or *Osr1*, both of which are required for development and maintenance of the embryonic gonad [28–30]. We found that, in the absence of either *Wt1* or *Osr1*, GATA4 is expressed in the coelomic epithelium, and the genital ridge is initially formed, although its growth is severely retarded and degeneration ensues (S3 and S4 Figs). In both *Wt1* KO and *Osr1* KO embryos, we observed DAZL expression in germ cells that migrated to the GATA4-expressing cells of the genital ridge, indicating that the association of PGCs with the nascent genital ridge is sufficient for licensing (S3 and S4 Figs). Taken together, our findings demonstrate that genital ridge formation is both necessary and sufficient to induce expression of DAZL in newly arrived PGCs.

### Germ cells in cultured urogenital ridges from *Gata4* cKO embryos retain PGC markers and fail to express GCC markers

**Fig 3:**
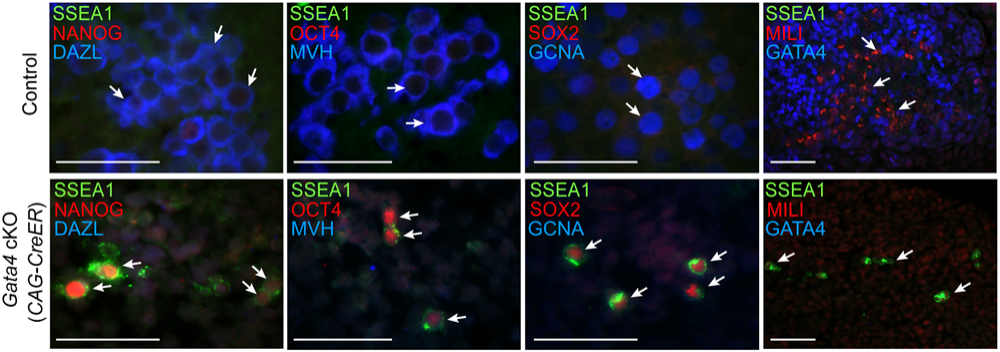
Germ cells in *Gata4* cKO embryos retain characteristics of PGCs. Immunofluorescent staining for PGC and GCC marker proteins in transverse sections of control (*Gata4* ^*+/flox*^ *)* and *Gata4* cKO (*CAG-CreER*) urogenital ridge cultures on a mixed genetic background. Arrows indicate representative germ cells. Scale bars: 50 μm.

Having found that germ cells in *Gata4* cKO embryos do not express DAZL and MVH, we wondered if these cells fail to transition into GCCs and instead retain characteristics of PGCs, such as continued expression of the pluripotency gene program and inability to sexually differentiate. Because *Gata4* cKO embryos die between E11.5-E12.0, we investigated subsequent germ cell development in urogenital ridge (UGR) cultures. The UGR was dissected from E11.5 control and *Gata4* ubiquitous cKO embryos, cultured for 3 days, and subjected to paraffin sectioning and immunofluorescent staining. In control UGR cultures (n = 5; 3 XX and 2 XY), all germ cells that we examined expressed markers seen in GCCs and sexually differentiating germ cells, including DAZL, MVH, GCNA, and MILI (Fig. 3). In addition, these germ cells switched off pluripotency markers, including NANOG, OCT4, SOX2, and SSEA1. In contrast, all germ cells in *Gata4* cKO UGR cultures (n = 4; 2 XX and 2 XY) retained an expression program similar to that of PGCs, with NANOG, OCT4, SOX2, and SSEA1 being expressed (Fig. 3). Conversely, markers of GCCs and sexually differentiating germ cells, including DAZL, MVH, GCNA and MILI, were not expressed in cultured UGRs from *Gata4* cKO embryos.

To confirm that the conversion of PGCs to GCCs depends upon *Gata4* function in somatic tissues and not the germline, we studied similar UGR cultures generated using the soma-specific Cre line, *Wt1-CreER*; *Osr1-CreER*. After 3 days of UGR culture, we observed that germ cells in controls expressed both DAZL and MVH, whereas germ cells in *Gata4* soma-specific cKO tissues expressed neither marker, and instead retained expression of the pluripotency marker SSEA1 (S5 Fig). These results indicate that the genital ridge is indispensable for licensing of PGCs to GCCs.

### Germ cells in *Gata4* cKO embryos fail to enter meiosis

**Fig 4:**
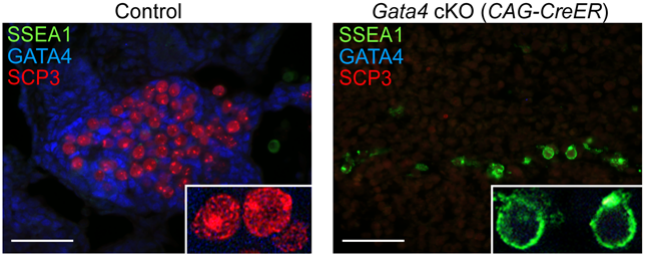
Germ cells in *Gata4* cKO embryos do not enter meiosis. Immunofluorescent staining for SSEA1, SYCP3, and GATA4 proteins in transverse sections of control (*Gata4* ^*+/flox*^ *)* and *Gata4* cKO (*CAG-CreER*) urogenital ridge cultures on a mixed genetic background. Inset shows higher magnification of cells. Scale bars: 50 μm.

A key functional characteristic that distinguishes GCCs from PGCs is their ability to enter meiosis. To investigate whether germ cells from *Gata4* cKO embryos are able to enter meiosis, we performed immunostaining on sections of cultured UGRs for SYCP3 and SSEA1 expression. We found that in control UGR cultures (n = 5 cultures), germ cells that expressed GCC markers (Fig. 3) showed SYCP3 assembly onto chromosomes—a characteristic of prophase of meiosis I—while the pluripotency marker SSEA1 was not detectable (Fig. 4). In contrast, germ cells in *Gata4* cKO UGR cultures (n = 4 cultures) expressed neither GCC markers (Fig. 3) nor SYCP3 (Fig. 4). Instead, they continued to express SSEA1, a marker of PGC identity. We conclude that germ cells in *Gata4* cKO UGR cultures were not competent to enter meiosis, functionally validating the earlier evidence that these cells had not become GCCs.

## Discussion

**Fig 5:**
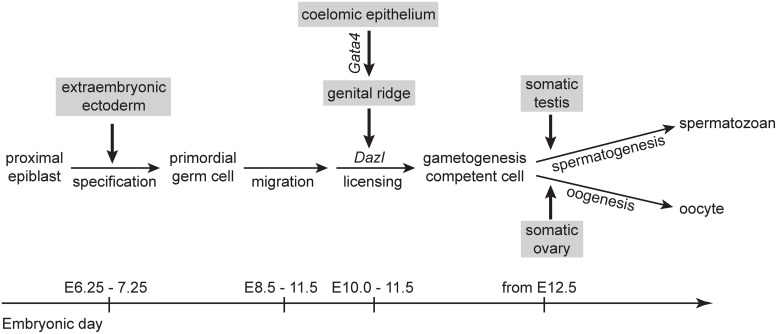
A proposed model for somatic induction of germ cell differentiation, in three steps. 1) Germ cell specification induced by signals, such as BMP4, from extraembryonic ectoderm [53,54]; 2) germ cell licensing induced by the genital ridge, which arises from the coelomic epithelium following *Gata4* expression; and 3) GCCs embark on either spermatogenesis or oogenesis in response to cues from somatic testis or ovary, respectively [12,13].

We have identified a previously unrecognized role of the genital ridge in germ cell development, prior to sex determination. We show that germ cell licensing—the transition of PGCs to GCCs—is induced by cues from the genital ridge (Fig. 5). If the genital ridge is not formed due to loss of the somatic transcription factor *Gata4*, germline cells remain at the PGC stage, failing to become GCCs and lacking the competence to undergo sexual differentiation and initiate meiosis. Thus, we provide genetic evidence that the transition of PGCs into meiotic germ cells is not a purely cell-autonomous process and is instead dependent on the somatic gonad.

The genital ridge-dependent induction of germ cell licensing is compatible with the observation that ectopic germ cells in the adrenal gland also enter meiosis [19]. Given that the genital ridge and adrenal gland share a common precursor, the adrenogonadal primordium [24,25], it is plausible that these two organs produce the same factors required to induce licensing and meiosis. Our finding also helps explain previous studies in which PGCs isolated from E10.5 embryos were shown to initiate meiosis in culture [13,22,23]. Because genital ridge formation is initiated as early as E10.0 [2], it is likely that a portion of E10.5 germ cells had already been exposed to the genital ridge environment. These licensed germ cells, therefore, had acquired meiotic competence by E10.5, before being cultured.

We have previously shown that *Dazl* is required for licensing of PGCs to GCCs in mouse embryos from an inbred C57BL/6 genetic background [11]. The present study extends our understanding of germ cell licensing for gametogenesis. We now appreciate that germ cell licensing is induced by the soma; indeed, the soma induces expression in germ cells of at least two key factors, DAZL and MVH, independently. Our findings also indicate that germ cell licensing depends upon the genital ridge in embryos of either mixed (Figs 2–4) or C57BL/6 genetic background (S2 and S5 Figs). The molecular mechanism by which the genital ridge regulates germ cell licensing requires further exploration.

We propose that the genital ridge—a somatic structure—induces germ cell licensing. However, we cannot formally exclude the possibility that the failure of licensing in germ cells of *Gata4* cKO embryos is a secondary effect of other activities at the genital ridge. The genital ridge has previously been shown to regulate germ cell motility, as PGCs become nonmotile after arriving at the genital ridge [18,31]. The genital ridge also produces factors that stimulate PGC proliferation [28,32,33]. Although germ cells colonize the coelomic epithelium in similar numbers in control and *Gata4* cKO embryos at E10.3 [2], *Gata4* cKO embryos display a reduced number of germ cells at E11.5, likely due to the absence of genital ridge-derived factors that stimulate proliferation. It is unlikely that this reduction in germ cell numbers is responsible for the licensing defects observed in the *Gata4* cKO, given that other mutants with reduced germ cell number display qualitatively normal germ cell development in males [34,35]. Similarly, we cannot yet exclude the possibility that systemic defects (e.g., in the gut, liver, or heart) contribute to the failure of germ cell licensing observed in the *Gata4* cKO. The generation of a genital ridge-specific Cre mouse will be required to rule this out.

We can now reconstruct the series of events in soma and germline (Fig. 5) that result in post-migratory PGCs initiating meiosis in the female fetal gonad, in an anterior-to-posterior (A-P) wave. In the soma, *Gata4* expression initiates the transformation of the coelomic epithelium (on the ventromedial surface of the mesonephros) into the genital ridge, in an anterior-to-posterior (A-P) wave [2]. Concurrently, PGCs migrate to the developing genital ridge as early as the monolayer stage. Based on our findings—that *Dazl* is expressed in germ cells in an A-P progression (Fig. 1), and that this expression is dependent upon the genital ridge (Fig. 2)—we propose that the progressive A-P development of the genital ridge induces *Dazl* expression and licensing in a similar A-P wave. Upon expression of DAZL, GCCs acquire the competence to interpret retinoic acid as a meiosis-inducing signal [36,37], and then express *Stra8*, the gene required for meiotic initiation [15,38], along with *Rec8* [39] and *Dmc1*—all in an A-P manner [40,41].

Licensing for gametogenesis constitutes a major transition during early germ cell development, allowing PGCs to acquire competence for sexual differentiation and gametogenesis. Our results indicate that the genital ridge triggers germ cell licensing. Thus, PGCs undergo licensing upon their arrival at the genital ridge, ensuring that gametogenesis occurs at the correct time and place.

## Materials and Methods

### Mice

All experiments involving mice were approved by the Committee on Animal Care at the Massachusetts Institute of Technology. The following mice were obtained from Jackson Laboratory (Stock Numbers 008194, 004682, 009061, 009387, 010912 and 002332): *Gata4* ^*flox/+*^ [42]; *CAG-CreER* (ubiquitously expressed) [43]; *Osr1* ^*eGFP-CreERt2/+*^ (somatically expressed) [44]; *Osr1* ^*tm1Jian/+*^ [30]; *Wt1* ^*CreERt2/+*^ (somatically expressed) [45]; and *Wt1* ^*tm1Jae/+*^[29], respectively. In some cases (as described in the text), these mice were backcrossed to the C57BL/6 strain (Taconic Farms) for at least 10 generations. *Gata4* cKO embryos were generated by mating *Gata4* ^*flox/flox*^ females with *Gata4* ^*+/∆*^ males carrying the indicated *CreER*. Where applicable, *Gata4* ^*+/flox*^ littermate embryos were used as controls. Tamoxifen (Sigma) was dissolved in corn oil (Sigma) at a concentration of 30 mg/ml. Dams were injected intraperitoneally at 8.75 days postcoitum with a single shot of tamoxifen (4 mg/40 g body weight) to induce excision of the floxed *Gata4* allele. The injection scheme was optimized for maximum embryo survival and *Gata4* excision efficiency [2]. Embryos were collected at the indicated time and immediately genotyped by PCR according to protocols from the Jackson Laboratory website.

### Single-molecule fluorescence *in situ* hybridization

Whole E9.5 embryos or urogenital ridges dissected from E11.5 embryos on C57BL/6 genetic background were fixed 2 hours at 4°C in 4% paraformaldehyde, equilibrated in 30% sucrose/4% paraformaldehyde in PBS overnight, and frozen and stored in OCT (Tissue-Tek) at -80°C before cryosectioning (8 μm thick). Probes were synthesized and hybridization performed as previously described [26]. Probes to *Dazl* and *Oct4* transcripts were conjugated to Cy5, A594, or TMR. AlexaFluor 488-conjugated anti-SSEA1 (560271, BD Biosciences) was added to the hybridization to label germ cells. Images were taken with a Nikon Ti-E inverted fluorescence microscope equipped with a 100x oil-immersion objective and a Photometrics Pixis 1024B CCD camera. We recorded stacks of images (z spacing 0.3 μm) at adjacent x-y positions covering the entire A-P length of the genital ridge. Images were stitched based on stage coordinates, and stitching coordinates were optimized locally by cross-correlation. Data analysis was performed in MATLAB (MathWorks) using custom-written code. Individual transcript molecules were identified and counted semi-automatically as previously described [26]. Individual germ cells were identified and outlined manually using a combination of SSEA1 expression and DAPI morphology. *Dazl* probe sequences used in this study are listed in S1 Table. *Oct4* probe sequences were published elsewhere [46].

### Urogenital ridge (UGR) cultures

Urogenital ridges, comprised of genital ridges, mesonephroi, primitive kidneys, and dorsal aorta, were dissected from E11.5 control and *Gata4* cKO embryos. The dissected UGRs were cultured on agar blocks, as previously described [47], for 3 days in Dulbecco’s modified Eagle’s medium (DMEM) containing 10% fetal bovine serum, non-essential amino acids, glutamine and penicillin/streptomycin. In this culture condition, as reported previously [13,23,48,49], E11.5 XY germ cells (which are sexually bipotential) develop toward the oogenic pathway, in part due to impaired testis cord formation and the presence of meiosis-inducing factors in the culture medium.

### Immunofluorescence

Immunofluorescent staining of embryonic sections was carried out as described previously [50]. Briefly, whole embryos or cultured urogenital organs were fixed at 4°C overnight in 4% paraformaldehyde, paraffin embedded, and sectioned. Slides were then dewaxed, rehydrated, and antigen-retrieved by microwaving in citrate buffer (10mM sodium citrate, 0.05% Tween 20, pH6.0). After blocking, slides were incubated with primary antibodies at 4°C overnight. Slides were then incubated with donkey secondary antibodies conjugated to FITC, Rhodamine Red X or DyLight 649 (Jackson ImmunoResearch) and mounted with ProLong Gold Antifade reagent with DAPI (Life Technologies).

Primary antibodies against GATA4 (sc-25310, Santa Cruz Biotechnology), DAZL (ab34139, Abcam), SSEA1 (MAB4301, Millpore), MVH (AF2030, R&D Systems), GCNA (a gift from George Enders, University of Kansas Medical Center, Kansas City, KS) [51], SOX2 (ab97959, Abcam), NANOG (IHC-00205, Bethyl Laboratories), OCT4 (560186, BD), MILI (a gift from Gregory J. Hannon, Cold Spring Harbor Laboratory, Cold Spring Harbor, NY) [52], and SYCP3 (sc-33195, Santa Cruz Biotechnology) were used in the study.

## Supporting Information

S1 Fig Post-migratory, but not migratory, germ cells express *Dazl*. (A) Immunofluorescent staining for SSEA1 and smFISH for *Dazl* and *Oct4* at E9.5. *Dazl* expression was not detectable in migratory PGCs, while *Oct4* was. Individual germ cells are outlined by dashed lines. (B) Representative E11.5 whole-gonad image of *Dazl* and *Oct4* transcript molecules marked by smFISH. *Dazl* transcript counts are higher in anterior region than in posterior region. *Dazl* (pseudocolored in blue, upper panel) and *Oct4* (pseudocolored in red, lower panel) mRNA molecules were overlaid with DAPI staining (white) and identified after raw images were subject to image analysis filtering. Raw images prior to filtering are displayed in magnified images (middle panels), where mRNA molecules are visible as white dots within individual germ cells (outlined by dashed lines).
(TIF)

S2 Fig Germ cells in *Gata4* cKO (soma-specific Cre) embryos do not express DAZL or MVH. Immunofluorescent staining for SSEA1, DAZL, MVH, and GATA4 in transverse sections of control and *Gata4* cKO (*Osr1* ^*CreER*^) embryos on a C57BL/6 genetic background. Nuclei counterstained with DAPI (blue). Scale bars: 50 μm.
(TIF)

S3 Fig Germ cells in *Wt1*-deficient genital ridges express DAZL. Genital ridge formation is initiated in *Wt1* KO embryos (a gift from Kenneth H. Albrecht), but growth is severely retarded and degeneration ensues. Immunofluorescent staining of longitudinal sections from wildtype or *Wt1* KO urogenital regions shows that PGCs at the genital ridge (GATA4-positive, blue) express DAZL (red, arrows). Yellow dashed lines outline the genital ridge. Autofluorescent red blood cells are indicated (asterisk). gr, genital ridge. Scale bars: 50 μm.
(TIF)

S4 Fig Germ cells in *Osr1*-deficient genital ridges express DAZL. Genital ridge formation is initiated in *Osr1* KO embryos, but complete degeneration occurs by E15.5 [30]. (A) Immunohistochemical staining for GATA4 in cross-sections of wildtype and *Osr1* KO embryos at E11.5. Genital ridge formation is initiated in *Osr1* KO embryos, but growth is restricted. Inset shows higher magnification of genital ridge. (B) Immunofluorescent staining for SSEA1, DAZL, and GATA4 in cross-sections of wildtype and *Osr1* KO urogenital regions. Representative germ cells positive for DAZL are indicated by arrows. Yellow dashed lines outline the genital ridge. a, dorsal aorta; gr, genital ridge; m, mesentery. Scale bars: 50 μm.
(TIF)

S5 Fig Germ cells in *Gata4* cKO (soma-specific Cre) cultured UGRs do not express DAZL or MVH. Immunofluorescent staining for SSEA1, DAZL, MVH, and 5-methyl-cytosine (meC) in transverse sections of control and *Gata4* cKO (*Wt1* ^*CreER*^;*Osr1* ^*CreER*^) cultured UGRs (on a C57BL/6 genetic background). Nuclei counterstained with DAPI (blue). Inset shows higher magnification of germ cells. Scale bars: 50 μm.
(TIF)

S1 Table *Dazl* probe sequences. Probe sequences used for smFISH analysis of *Dazl* expression.
(DOCX)
