# Supplementary figures and images for: Licensing of Primordial Germ Cells for Gametogenesis Depends on Genital Ridge Signaling
Source: PLoS Genet. 2015 Mar 4;11(3):e1005019. doi: 10.1371/journal.pgen.1005019 (PMC4349450; doi:10.1371/journal.pgen.1005019)

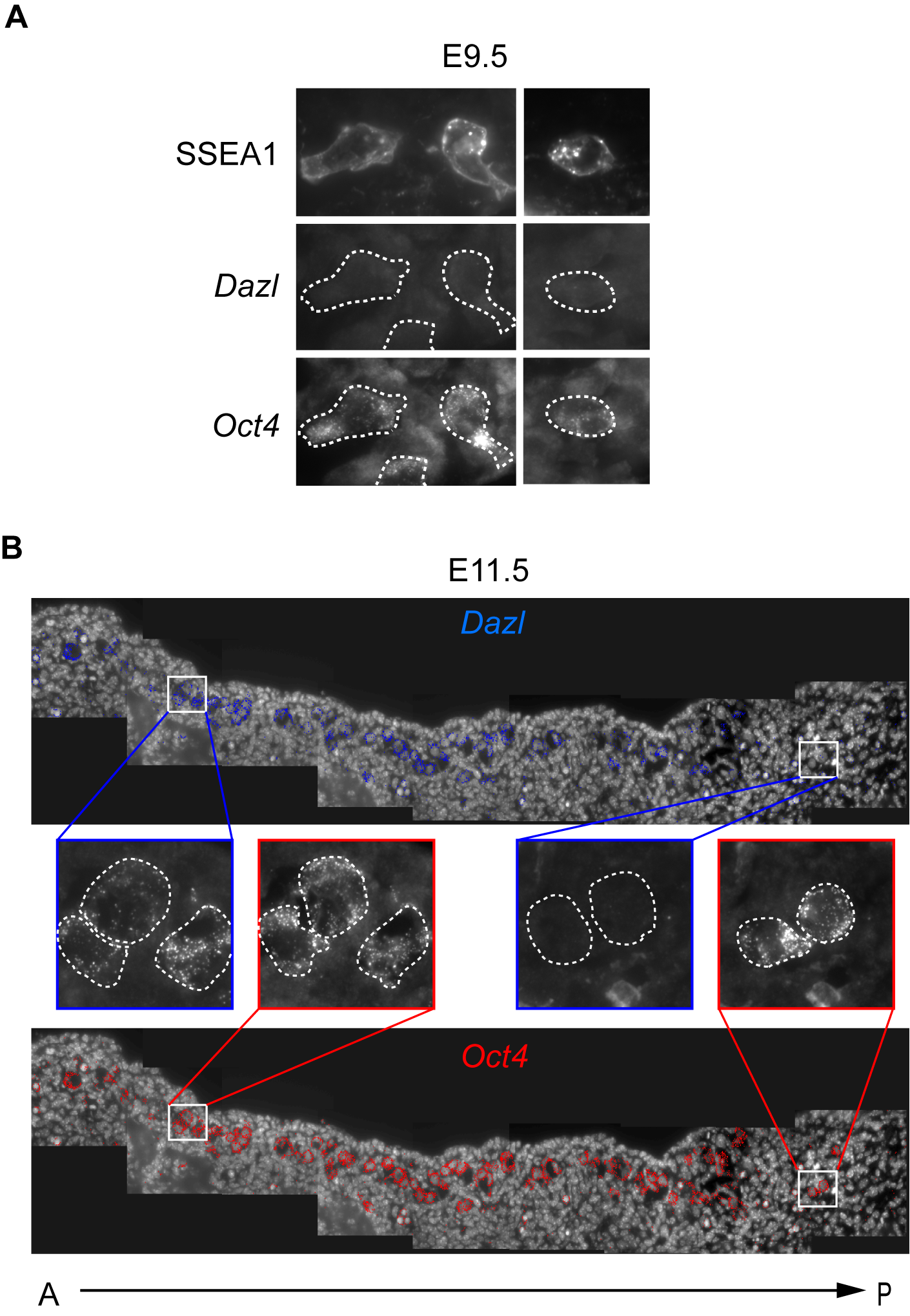

Supplement: S1 Fig — (A) Immunofluorescent staining for SSEA1 and smFISH for Dazl and Oct4 at E9.5. Dazl expression was not detectable in migratory PGCs, while Oct4 was. Individual germ cells are outlined by dashed lines. (B) Representative E11.5 whole-gonad image of Dazl and Oct4 transcript molecules marked by smFISH. Dazl transcript counts are higher in anterior region than in posterior region. Dazl (pseudocolored in blue, upper panel) and Oct4 (pseudocolored in red, lower panel) mRNA molecules were overlaid with DAPI staining (white) and identified after raw images were subject to image analysis filtering. Raw images prior to filtering are displayed in magnified images (middle panels), where mRNA molecules are visible as white dots within individual germ cells (outlined by dashed lines). (TIF) [file pgen.1005019.s001.tif]

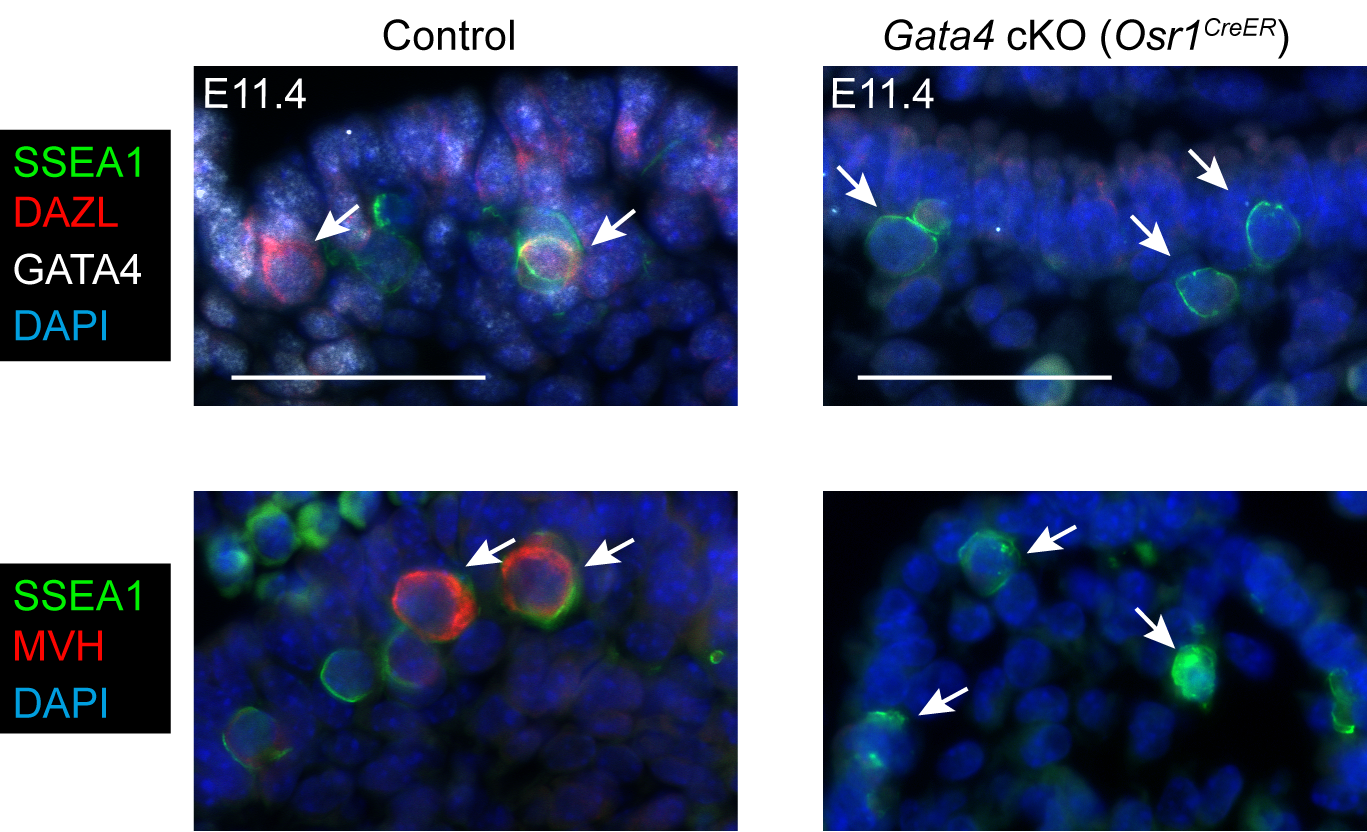

Supplement: S2 Fig — Immunofluorescent staining for SSEA1, DAZL, MVH, and GATA4 in transverse sections of control and Gata4 cKO (Osr1 CreER) embryos on a C57BL/6 genetic background. Nuclei counterstained with DAPI (blue). Scale bars: 50 μm. (TIF) [file pgen.1005019.s002.tif]

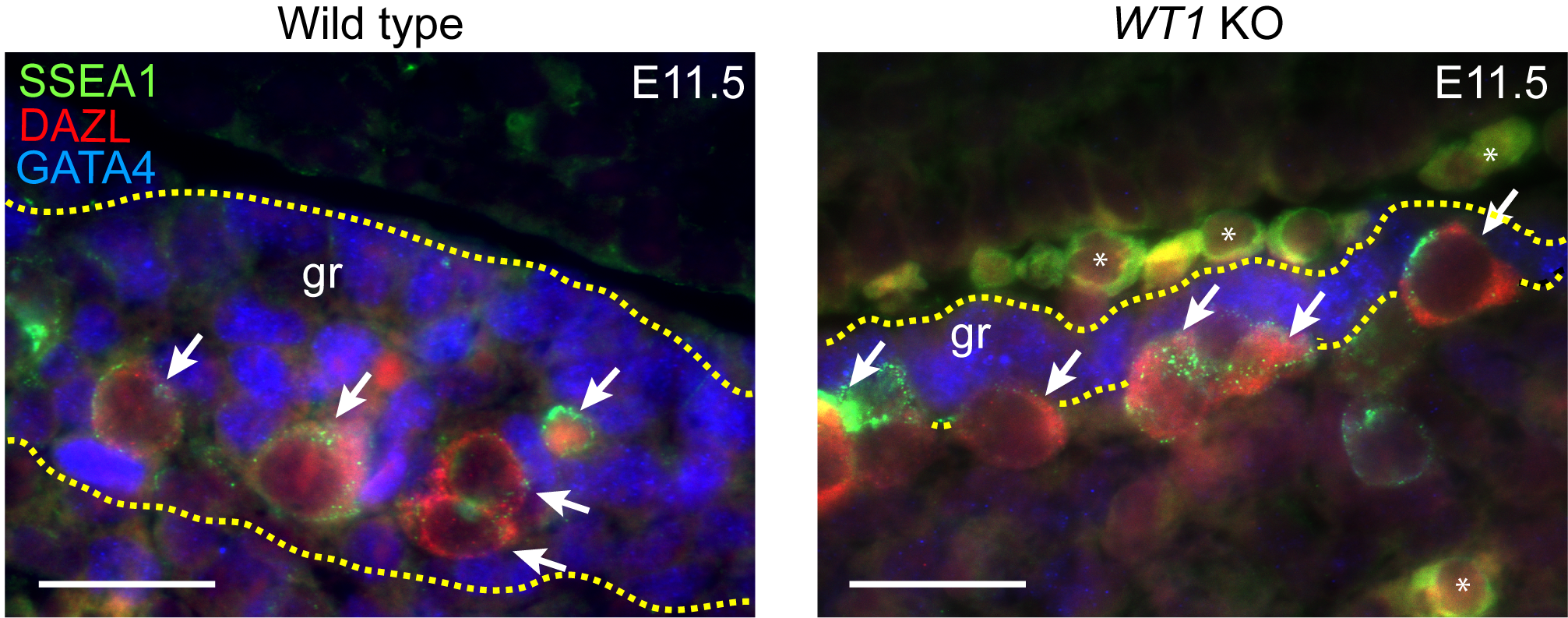

Supplement: S3 Fig — Genital ridge formation is initiated in Wt1 KO embryos (a gift from Kenneth H. Albrecht), but growth is severely retarded and degeneration ensues. Immunofluorescent staining of longitudinal sections from wildtype or Wt1 KO urogenital regions shows that PGCs at the genital ridge (GATA4-positive, blue) express DAZL (red, arrows). Yellow dashed lines outline the genital ridge. Autofluorescent red blood cells are indicated (asterisk). gr, genital ridge. Scale bars: 50 μm. (TIF) [file pgen.1005019.s003.tif]

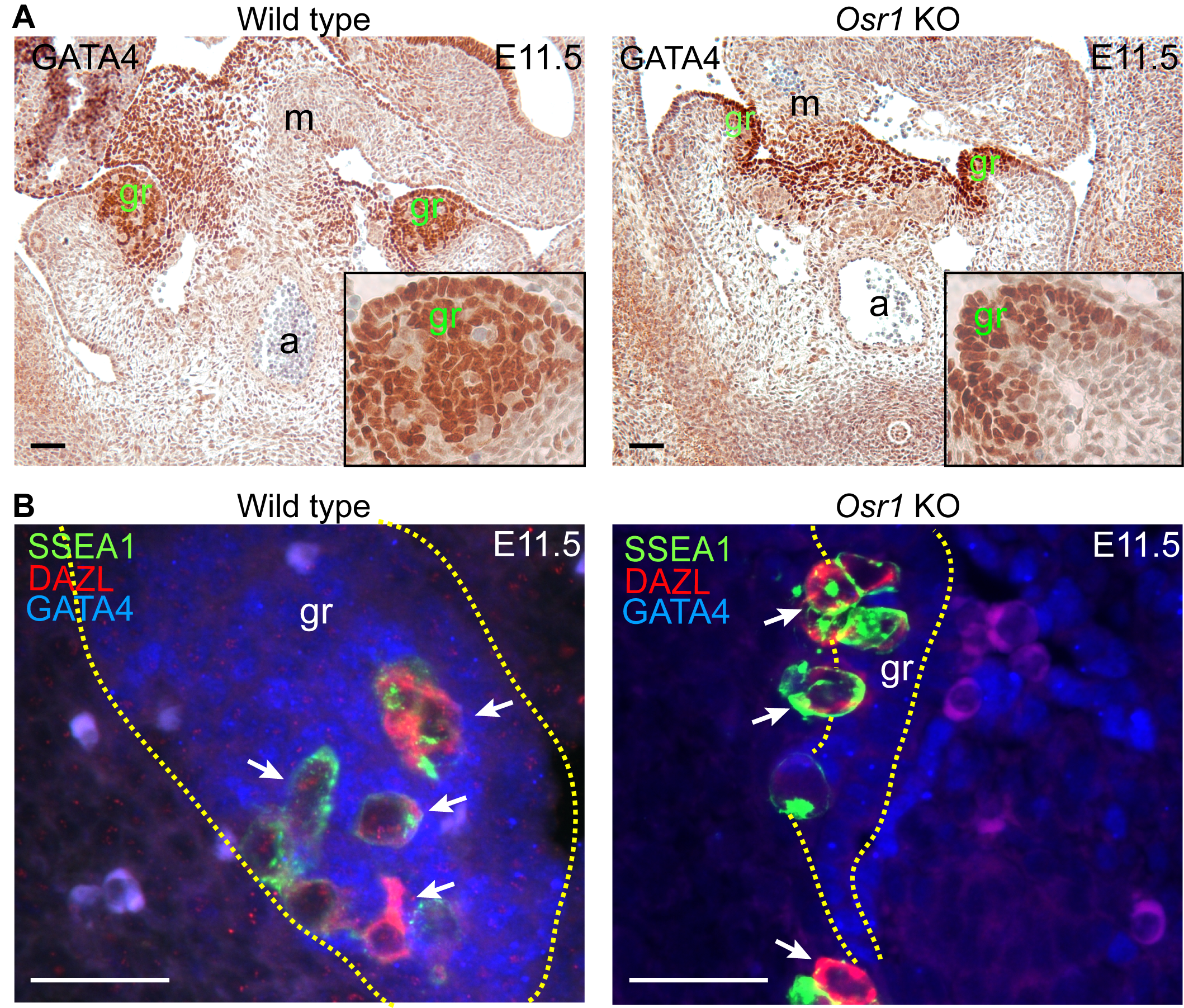

Supplement: S4 Fig — Genital ridge formation is initiated in Osr1 KO embryos, but complete degeneration occurs by E15.5 [30]. (A) Immunohistochemical staining for GATA4 in cross-sections of wildtype and Osr1 KO embryos at E11.5. Genital ridge formation is initiated in Osr1 KO embryos, but growth is restricted. Inset shows higher magnification of genital ridge. (B) Immunofluorescent staining for SSEA1, DAZL, and GATA4 in cross-sections of wildtype and Osr1 KO urogenital regions. Representative germ cells positive for DAZL are indicated by arrows. Yellow dashed lines outline the genital ridge. a, dorsal aorta; gr, genital ridge; m, mesentery. Scale bars: 50 μm. (TIF) [file pgen.1005019.s004.tif]

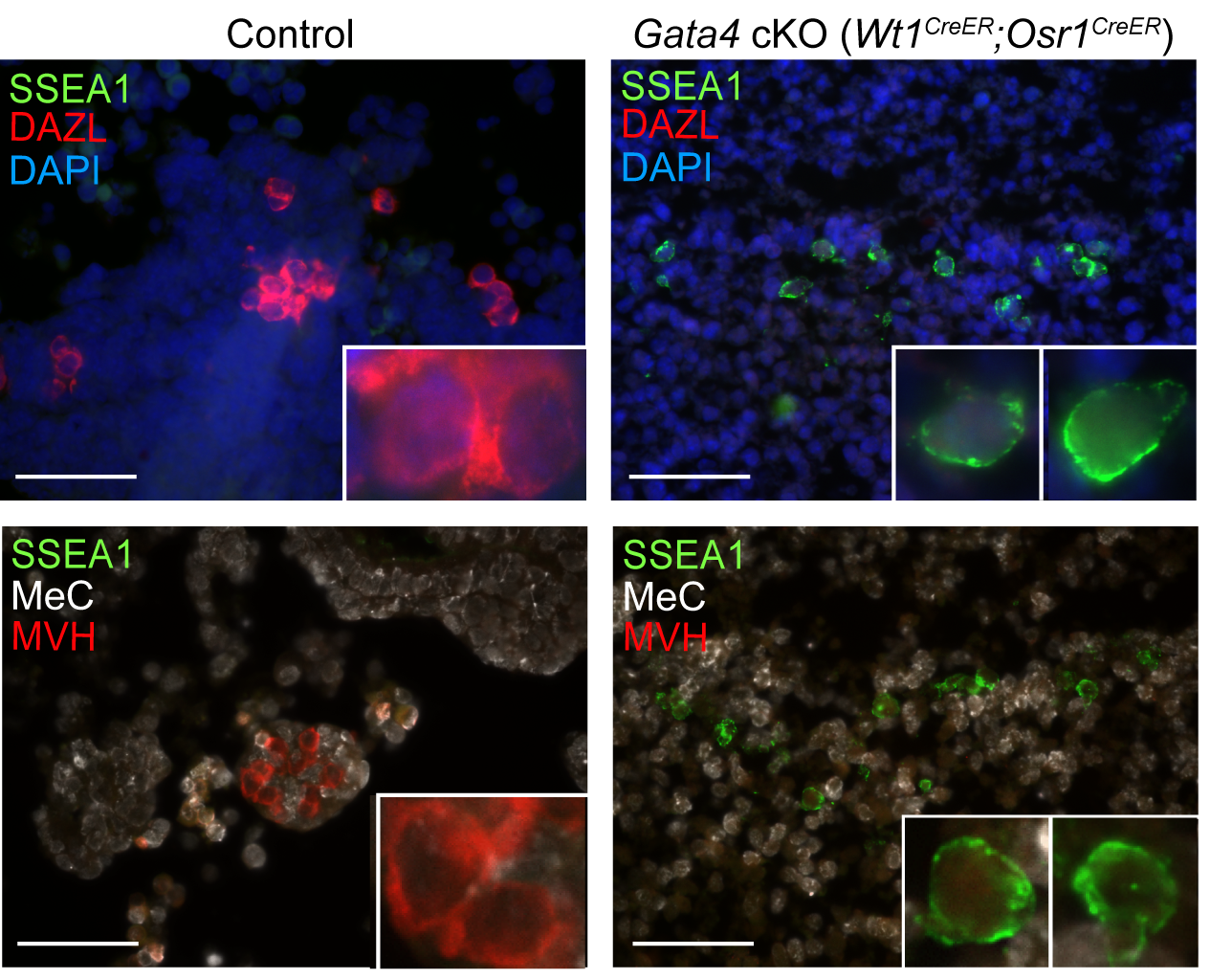

Supplement: S5 Fig — Immunofluorescent staining for SSEA1, DAZL, MVH, and 5-methyl-cytosine (meC) in transverse sections of control and Gata4 cKO (Wt1 CreER;Osr1 CreER) cultured UGRs (on a C57BL/6 genetic background). Nuclei counterstained with DAPI (blue). Inset shows higher magnification of germ cells. Scale bars: 50 μm. (TIF) [file pgen.1005019.s005.tif]
