# Supplementary material for: Licensing of Primordial Germ Cells for Gametogenesis Depends on Genital Ridge Signaling
Source: PLoS Genet. 2015 Mar 4;11(3):e1005019. doi: 10.1371/journal.pgen.1005019 (PMC4349450; doi:10.1371/journal.pgen.1005019)
Supplement: S1 Table — Probe sequences used for smFISH analysis of Dazl expression. (DOCX) [file pgen.1005019.s006.docx]

Table S1 *Dazl* probe sequences

| No. | Sequence (5’ to 3’) | No. | Sequence (5’ to 3’) |
| --- | --- | --- | --- |
| 1 | tcagaagttgtggcagacat | 25 | gcagctgatatccagtgatg |
| 2 | tggagacagctgaatttgga | 26 | ggtggcatctggtagttata |
| 3 | tgaagactgagtgctggcct | 27 | aactcctctgctctccagca |
| 4 | atccttgacttgtggttgct | 28 | gtataagccggaggtataac |
| 5 | attttgccttctggcaaaac | 29 | actgcagtggtagttaacag |
| 6 | caacaaaaacggtgtttggc | 30 | tatcagctcctggatcaact |
| 7 | tccatcctaacatcaattcc | 31 | actgaacattcattgggcaa |
| 8 | gaaactcctgatttcggttt | 32 | agaagctggagcagcatcat |
| 9 | ttactgagccatatctggca | 33 | acttcttttgcgggccattt |
| 10 | tcagtgattatcttcacttc | 34 | tgtctgtatgcttcggtcca |
| 11 | agcccttcgacacaccagtt | 35 | ggttaaacagacaagagacc |
| 12 | ttataaaatgagacaaatcc | 36 | gagtttctcagtctgttctc |
| 13 | tatcttctgcacatccacgt | 37 | gtagtcatcttgagtaacaa |
| 14 | ggaaatttatctgtgattct | 38 | gatgtactcttttatccttg |
| 15 | cccagtttcagctttttacc | 39 | actgcccgacttcttctgaa |
| 16 | attttgtttcctgattgcag | 40 | gcagagatgatcagatttaa |
| 17 | gctgcacatgataagtacat | 41 | ttcaaaaccaacaaccccct |
| 18 | ggattaaaaatcaaaggacg | 42 | gaaagtctgagtgatttgtc |
| 19 | tctggaactgtggtggagga | 43 | gaacacaaaaccagtttcta |
| 20 | gcatttggactactccaaac | 44 | caactaaaagtccttacggt |
| 21 | tggaggctgcatgtaagtct | 45 | cctgacctttcaaaataggt |
| 22 | gagtgataggattcatcatg | 46 | ggtacctgttgagaagtgaa |
| 23 | ggaggatatgcctgaacata | 47 | gtggcaaatgtttagacaaa |
| 24 | ctgaactggtgaacttggat | 48 | gcagttctaaaaattctgct |
